# The Effector Protein CgNLP1 of *Colletotrichum gloeosporioides* Affects Invasion and Disrupts Nuclear Localization of Necrosis-Induced Transcription Factor HbMYB8-Like to Suppress Plant Defense Signaling

**DOI:** 10.3389/fmicb.2022.911479

**Published:** 2022-06-13

**Authors:** Guangyong Yang, Jie Yang, Qiwei Zhang, Wenfeng Wang, Liping Feng, Li Zhao, Bang An, Qiannan Wang, Chaozu He, Hongli Luo

**Affiliations:** ^1^Hainan Key Laboratory for Sustainable Utilization of Tropical Bioresources, College of Tropical Corps, Hainan University, Haikou, China; ^2^Hainan Yazhou Bay Seed Laboratory, Sanya Nanfan Research Institute of Hainan University, Sanya, China

**Keywords:** *Colletotrichum gloeosporioides*, CgNLP1, pathogenic mechanism, *Hevea brasiliensis*, HbMYB8-like

## Abstract

Fungi secrete numerous effectors to modulate host defense systems. Understanding the molecular mechanisms by which fungal effectors regulate plant defense is of great importance for the development of novel strategies for disease control. In this study, we identified necrosis- and ethylene-inducing protein 1 (Nep1)-like protein (NLP) effector gene, *CgNLP1*, which contributed to conidial germination, appressorium formation, invasive growth, and virulence of *Colletotrichum gloeosporioides* to the rubber tree. Transient expression of CgNLP1 in the leaves of *Nicotiana benthamiana* induced ethylene production in plants. Ectopic expression of CgNLP1 in *Arabidopsis* significantly enhanced the resistance to *Botrytis cinerea* and *Alternaria brassicicola*. An R2R3 type transcription factor HbMYB8-like of rubber tree was identified as the target of CgNLP1.HbMYB8-like, localized on the nucleus, and induced cell death in *N. benthamiana*. CgNLP1 disrupted nuclear accumulation of HbMYB8-like and suppressed HbMYB8-like induced cell death, which is mediated by the salicylic acid (SA) signal pathway. This study suggested a new strategy whereby *C. gloeosporioides* exploited the CgNLP1 effector to affect invasion and suppress a host defense regulator HbMYB8-like to facilitate infection.

## Introduction

To successfully infect and cause disease, phytopathogenic fungi need to form intimate associations and maintain constant communication with a susceptible host, and this communication can be achieved through the proteins, enzymes, and metabolites secreted by phytopathogenic fungi (Heard et al., 2015). Effector proteins are small cysteine-rich proteins secreted by pathogens and play roles in virulence and the interaction between plants and pathogens. According to the innate immunity theory, plants have evolved two strategies to detect pathogens: one is the recognition of conserved microbial elicitors called pathogen-associated molecular patterns (PAMPs) by receptor proteins called pattern recognition receptors (PRRs) on the external face of the host cell, which leads to PAMP-triggered immunity (PTI); another one is the recognition of pathogen virulence molecules called effectors by plant intracellular receptors called R protein, which leads to effector-triggered immunity (ETI) (Dodds and Rathjen, 2010).

The necrosis- and ethylene-inducing protein 1 (Nep1)-like protein (NLP) is an important effector family and is named after the necrosis and ethylene-inducing protein (NEP1) first identified from the culture filtrate of *Fusarium oxysporum* f.sp. *erythroxyli* (Bailey, 1995; Chen et al., 2018). NLPs widely distributed in oomycetes, fungi, and bacteria and shared a conserved necrosis-inducing phytophthora protein (NPP1) domain, typically containing a GHRHDWE heptapeptide motif that was crucial for toxicity (Gijzen and Nürnberger, 2006; Lenarči et al., 2017). Based on the molecular structures and sequence comparison analysis, NLPs were divided into three phylogenetic group types, namely, type I, type II, and type III (Oome et al., 2014; Levin et al., 2019; Seidl and Ackerveken, 2019). Type I NLPs contained a conserved disulfide bond and were found predominately in plant microorganisms. Compared with type I, type II had a second conserved disulfide bond and an additional putative calcium-binding domain. Type III NLPs were different from the other types in the amino acid sequence of the N- and C-terminal portion, and there is still very minimal experimental data available (Oome et al., 2014; Lenarči et al., 2017). Based on the ability to induce necrosis, NLPs were divided into two groups (Schumacher et al., 2020). Group one was cytotoxic NLP proteins and group two was non-cytotoxic NLP proteins (Amsellem et al., 2002; Santhanam et al., 2013).

The members of group I as their names suggested were best known as a virulence factor for inducing necrosis and ethylene production in plant leaves (Bailey, 1995; Oome et al., 2014; Levin et al., 2019; Seidl and Ackerveken, 2019). MoNEP1, MoNLP2, and MoNEP4, which are three NLP proteins from the hemibiotrophic plant pathogenic fungus *Magnaporthe grisea*, induced cell death and the production of reactive oxygen species in *Nicotiana benthamiana* (Fang et al., 2017). Normally, the cytotoxic NLP proteins were expressed during the switch from biotrophic to necrotrophic lifestyle in hemibiotrophic pathogens (Alkan et al., 2015). BcNEP1 and BcNEP2, which are two paralogous NLPs from the necrotrophic plant pathogenic fungus *Botrytis cinerea*, caused necrosis in all dicotyledonous plant species tested (Schouten et al., 2008). In vascular wilt pathogen *Verticillium dahlia*, only two of the seven NLPs induced plant cell death, and only one of these two NLPs plays a role in vegetative growth and asexual reproduction in addition to their contribution to virulence (Santhanam et al., 2013). NLP-induced cell death was an active, light-dependent process that required HSP90 and interacts with a target site on the extracytoplasmic side of dicot plant plasma membranes (Qutob et al., 2006). Biochemical analyses had revealed that the target of NLP on plant membrane was sphingolipid-glycosyl inositol phosphorylated ceramide (GIPC), which consisted of an inositol phosphoceramide and a head group consisting of glucuronic acid and a variable number and form of terminal hexoses. When NLPs bind to the head group of GIPC in monocotyledons, the three terminal hexoses prevented NLPs from inserting into the lipid bilayers of cell membranes, while the GIPC head of dicotyledons only had two terminal hexoses, allowing NLPs to insert into cell membranes and causing cell necrosis (Van den Ackerveken, 2017). Recent research results showed that a plant-derived LRR-only protein NTCD4 promotes NLP-triggered cell death and disease susceptibility by facilitating oligomerization of NLP in *Arabidopsis* (Chen et al., 2021).

Group II was non-cytotoxic NLP proteins, which were often expressed during the very early stages of the infection or keep at rather low levels during the whole course of infection (Cabral et al., 2012; Dong et al., 2012; Schumacher et al., 2020). These non-cytotoxic NLPs also acted as triggers of plant innate immune responses, including posttranslational activation of mitogen-activated protein kinase activity, deposition of callose, production of nitric oxide, reactive oxygen intermediates, ethylene, phytoalexin camalexin, and cell death (Fellbrich et al., 2000; Kanneganti et al., 2006; Qutob et al., 2006; Rauhut et al., 2009; Villela-Dias et al., 2014; Seidl and Ackerveken, 2019). Ten different non-cytotoxic NLPs (HaNLPs) from biotrophic downy mildew pathogen *Hyaloperonospora arabidopsidis* did not cause necrosis but acted as potent activators of the plant immune system in *Arabidopsis thaliana*, and ectopic expression of HaNLP3 in *Arabidopsis* enhanced the resistance to *H. arabidopsidis* and activated the expression of a large set of defense-related genes (Oome et al., 2014). SsNEP2 was involved in fungal virulence by affecting ROS levels in *Sclerotinia sclerotiorum* and triggers host PTI as a PAMP to promote the necrotrophic lifestyle of *S. sclerotiorum* (Yang et al., 2022). Moreover, it was also reported that multiple cytotoxic NLPs carried a motif of 20 amino acid residues (nlp20), and nlp20 could trigger PTI by binding *in vivo* to a tripartite complex RLP23-SOBIR1-BAK1 (Böhm et al., 2014; Albert et al., 2015). Ectopic expression of RLP23 in potato (*Solanum tuberosum*) enhanced immunity to *Phytophthora infestans* and *S. sclerotiorum* (Albert et al., 2015).

*Colletotrichum* causes anthracnose on a wide variety of woody plants in tropical, subtropical, and temperate climates (Liang et al., 2021). In *Colletotrichum*, six NLP homologs were identified in the *Colletotrichum higginsianum* genome. Of them, ChNLP1 induced cell death when expressed transiently in *N. benthamiana* and was expressed specifically at the switch from biotrophy to necrotrophy, whereas ChNLP3 lacks necrosis-inducing activity and was expressed in appressoria before penetration (Kleemann et al., 2012). An effector NLP1 from *Colletotrichum orbiculare* induced necrosis in *N. benthamiana* and also possessed a MAMP sequence called nlp24, which triggered the ROS accumulation in leaf discs of *Arabidopsis* (Azmi et al., 2017). As one of the most important pathogenic species of *Colletotrichum, Colletotrichum gloeosporioides* is the dominant causal agent of rubber tree anthracnose and leads to serious loss of natural rubber production (Liu et al., 2018; Wang et al., 2018). However, less is known about the pathogenesis of *C. gloeosporioides* on a rubber tree. To elucidate the pathogenic mechanism of *C. gloeosporioides*, the candidate genes encoding effector were predicted in the genome of *C. gloeosporioides*, and one of them was named *CgNLP1*. In this study, we confirmed the contribution of CgNLP1 to fungal invasion, the virulence of *C. gloeosporioides* on the rubber tree, and the enhanced resistance of *Arabidopsis* to *B. cinerea* and *Alternaria brassicicola*. In addition, HbMYB8-like, an R2R3-type transcription factor (TF) localized on the nucleus and inducing necrosis, was first identified as the target of CgNLP1 in the rubber tree, and further studies showed that CgNLP1 could disrupt the nuclear translocation of HbMYB8-like and inhibit HbMYB8-like-induced cell death mediated by salicylic acid (SA) signaling. The functional interference of CgNLP1 on HbMYB8-like extended our new understanding of the pathogenic mechanism of *C. gloeosporioides*.

## Materials and Methods

### Biological Materials and Growth Conditions

*Colletotrichum gloeosporioides* strain was isolated from the leaves of *Hevea brasiliensis* with anthracnose. *B. cinerea* and *A. brassicicola* were provided courtesy of Tesfaye's lab. All fungal strains were grown on potato dextrose agar (PDA) at 28°C in the dark. *Hevea brasiliensis* (Reyan 7-33-97) was grown on the soil at 28°C. *Arabidopsis thaliana* Columbia ecotype and *N. benthamiana* were grown on soil under fluorescent light (200 μE·m^2^·s^−1^) at 22°C with 60% RH and a 12-h-light/12-h-dark cycle.

### RNA Isolation, cDNA Synthesis, PCR Amplification, and qRT-PCR

Fungal total RNA was extracted using the CTAB-LiCl method (Yang et al., 2020). Plant total RNA was extracted according to the instruction of the polysaccharide polyphenol plant total RNA extraction kit (Tiangen: DP441). The contaminating DNA was eliminated using RNase-free DNase, and the first-strand cDNA was synthesized using the Revert Aid First Strand cDNA Synthesis Kit (Thermo Fisher). Quantitative RT-PCR analysis was performed using ChamQ Universal SYBR qPCR Master Mix (Vazyme Biotech: Q711-02) with the LightCycler 96 System (Roche). The *Nicotiana tabacum actin-7* (*NtActin 7*) and *H. brasiliensis* 18S rRNA (*Hb18S*) were used as an endogenous control for normalization. The primers used for quantitative RT-PCR and PCR amplification are listed in Supplementary Table 1. Relative expression levels of target genes were estimated using the 2^−ΔΔCt^ method.

### Sequence Analysis of CgNLP1 and HbMYB8-Like

The amino acid sequences were deduced using DNAMAN software. Predictions of signal peptides were performed online using the SignalP 5.0 analysis tool (http://www.cbs.dtu.dk/services/SignalP/). The conserved domains were predicted using the SMART website (http://smart.embl-heidelberg.de/). The multiple alignments of amino acid sequences were performed using ESPript 3.0 (http://espript.ibcp.fr/ESPript/cgi-bin/ESPript.cgi) and GeneDoc 2.7.0. The bootstrap neighbor-joining phylogenetic tree was constructed using Clustal X 2.0 and MEGA 7.0.

### Generation of *CgNLP1* Knockout and Complementary Mutants

Based on the diagram of the *CgNLP1* knockout vector, the 5′ and 3′ flanking regions of *CgNLP1* were amplified from genomic DNA and ligated into the vector pCB1532 carrying the acetolactate synthase gene (SUR) cassette conferred resistance to chlorimuron ethyl (a sulfonylurea herbicide). For the complementation vector, the open read frame of *CgNLP1* fused with the 3X FLAG coding sequence was cloned into the vector harboring the promoter of *ToxA*, the terminator of nos, and the hygromycin phosphotransferase gene (HPH) (Supplementary Figure S2A). The fungal transformation was carried out as described in our previous study (Wang et al., 2017). The mutants were analyzed by PCR analyses. The primers used for *CgNLP1* amplifying and mutant diagnosis are listed in Supplementary Table 1.

### Ethylene Production Assays

CgNLP1 was transiently expressed in tobacco leaves. After Agrobacterium injection, each 0.2–0.4 g of tobacco leaves expressing CgNLP1 was immediately placed in different 20 ml sealed containers, and the ethylene contents were measured at different time points. A volume of 0.5 ml of ethylene released from tobacco leaves in each container was moved to a syringe for ethylene contents assay using a portable ethylene analyzer (GC-FID 8890, Agilent, USA). The ethylene evolution rate (μl/g∙h) was calculated using the formula PPm × L/W∙h (PPm: concentration of ethylene released from the sample to be tested, L: volume of injection bottle and volume of sample to be tested, W: weight of the sample to be tested (g), and h: sealing time). Ethylene standards (99.99% purity) were used for standard curve construction.

### Construction of *CgNLP1* Overexpression Lines in *Arabidopsis*

The vector pER8-*CgNLP1*-FLAG was constructed with an estradiol-inducible promoter and then transformed into *Agrobacterium tumefaciens* GV3101. The *Agrobacterium*-mediated flower dip method was used for *Arabidopsis* transformation of *CgNLP1*. T1 transgenic lines were screened with 30 mg/L hygromycin, and Western blot was used to confirm the positive transgenic lines after estradiol treatment. Appropriate total proteins from different lines were resolved on 10% polyacrylamide gels and transferred to a nitrocellulose membrane (Bio-Rad). Anti-FLAG monoclonal antibody (1:1,000, ab125243; Abcam) was used as a primary antibody to detect the expression of CgNLP1 in transgenic lines. Horseradish peroxidase-conjugated anti-mouse antibody was used as the secondary antibody, and the signal was detected using the ECL western detection kit (RPN2232; GE Healthcare).

### Disease Assays

For the pathogenicity test of *C. gloeosporioides* to rubber tree, conidia were harvested from mycelium grown on PDA (potato dextrose agar, Difco) medium for 10 days in a 28°C incubator and resuspended in a solution of PD (potato dextrose broth, Difco) liquid medium to a final concentration of 2 × 10^5^ conidia/ml. Then, 10 μl of the conidial suspensions were inoculated onto the wounded rubber tree variety 7-33-97 leaves at the “light green” stage. The inoculated leaves were kept in a moist chamber at 28°C under natural illumination for 4 days, and the disease symptoms were scored.

*Botrytis cinerea* and *A. brassicicola* disease assays were performed on detached leaves by drop inoculation. Both strains were cultured in a 2 × V8 solid medium and incubated at 22°C. Conidia of *B. cinerea* were collected and suspended in 1% Sabouraud maltose broth buffer (Difco) containing 0.05% (v/v) Tween-20, and the conidial density was adjusted to 2.5 × 10^5^ spores/ml before inoculation. Conidia of *A. brassicicola* were collected and suspended in water containing 0.05% (v/v) Tween-20, and the conidial density was adjusted to 5 × 10^5^ spores/ml before inoculation. In both cases, the droplets of 5 μl spore suspension above were inoculated on 4-week-old *Arabidopsis* leaves. The inoculated plants were kept under a transparent cover to maintain high humidity. The lesion diameters were measured to assess the levels of plant disease resistance. Each treatment contained three replicates of 9 leaves, and the entire experiment was repeated three times.

### Fungal Growth and Development Assay

The radial growth, conidiation, and appressorium formation assays were performed as described by Liang et al. (2021). For the vegetate growth assay, 5-mm-diameter mycelium disks were inoculated on potato dextrose agar medium and cultured for 6 days, the diameters of the colonies were recorded, and the growth rates were calculated. For the conidiation assay, conidia harvested from PDA culture plates were inoculated into 50 ml liquid CM medium to the final concentration of 10^4^/ml and then cultured at 28°C, 150 rpm, and the conidial numbers were calculated under a microscope after incubation for 2 days. For appressorium formation assays, conidial suspensions were placed on a nylon membrane and incubated at 28°C for 4 h of post-incubation, and the percentages of conidial germination and appressorium formation were determined under a microscope. The invasive growth assay was performed on the onion epidermis, which was sprayed with 2.5 × 10^5^/ml of conidia and placed on a PDA medium plate at 28°C for 12 h, and the invasion was observed under a microscope. The experiments were repeated three times, and at least 100 conidia were detected per replicate.

### Subcellular Localization and Bimolecular Fluorescence Complementation Assays

For subcellular localization of HbMYB8-like, the coding sequence of *HbMYB8-like* was inserted into the transient expression vector 35S-MCS-mScarlet to generate recombinant plasmid HbMYB8-like-RFP. The vector MEIL-RFP was used as a marker vector for plasma membrane and nuclear localization (Guy et al., 2013). For the bimolecular fluorescence complementation (BiFC) assay, the coding sequences of *CgNLP1* and *HbMYB8-like* were inserted into pSPYCE-YFP^C^ and pSPYNE-YFP^N^, respectively, to generate recombinant plasmids pSPYCE-*CgNLP1*-YFP^C^ and pSPYNE-*HbMYB8-like*-YFP^N^. The above constructs were verified by sequencing and then introduced into Agrobacterium strain GV3101, respectively. The Agrobacterium carrying HbMYB-like-RFP was expressed in *N. benthamiana* leaf tissue by agroinfiltration for subcellular localization. pSPYCE-*CgNLP1*-YFP^C^ and pSPYNE-*HbMYB8-like*-YFP^N^ were co-expressed in *N. benthamiana* leaf tissue for BiFC assay. The fluorescence distribution was observed using a laser confocal microscope (Leica TCS SP8).

### Phytohormone Treatment

Seedlings of Reyan7-33-97 were treated with 5 mM SA, 1 mM methyl jasmonate (MeJA), and 0.5 mM ethephon (ET) and placed in a 25°C greenhouse (Radojičić et al., 2018). After 0, 12, 24, and 48 h of following the treatment, leaves from seedlings were collected and quickly frozen in liquid nitrogen and then stored in a −80°C refrigerator for RNA extraction. Two leaves from each seedling were picked, and three seedlings were pooled as one biological replicate. Each seedling was harvested only once.

### Yeast Two-Hybrid Screens

Yeast two-hybrid assays were performed using the Matchmaker™ Gold Yeast Two-Hybrid System according to the manufacturer's instructions (Clotech: No.630489). The coding sequence of *CgNLP1* was amplified and cloned into pGBKT7 to generate DNA binding domain bait protein fusion. The cDNA libraries of *Hevea brasiliensis* were constructed according to the manufacturer's instructions (Clotech: PT4085-1). Interacting proteins were selected for selective medium lacking His, Leu, Trp, and Ade. The putative interactors were then tested by assaying for the lacZ reporter gene activation as described in the Clontech protocol. The plasmids from the positive clones were then isolated and reintroduced into the original yeast bait and control bait strains to verify interaction.

### GST Pull-Down Assays

The prokaryotic expression vectors, namely, pColdTM TF-CgNLP1 and pGEX-HbMYB8-like were constructed and transferred into *Escherichia coli* BL21 (DE3), respectively. The expression of the fusion proteins was performed as described in the product manuals (Beyotime Biotechnology: P2262). The cell lysate supernatants containing GST-HbMYB8-like and His-CgNLP1 fusion protein were incubated with GST binding gels at 4°C overnight and the supernatants containing GST + His-CgNLP1 as control. The pull-down reactions were analyzed by SDS-PAGE followed by Western blot using anti-His (M20001; Abmart) and anti-GST (ab111947; Abcam) antibodies.

### Trypan Blue Staining

Four-week-old tobacco leaves were infiltrated with *Agrobacterium tumefaciens* GV3101 harboring empty vector pEGAD-eGFP and recombinant plasmid pEGAD-*CgNLP1*-eGFP, respectively. Leaves were harvested 3 h after infiltration and stained for cell death using the Trypan Blue Staining Cell Viability Assay Kit (Beyotime Institute of Biotechnology, Haimen, China). For destaining, the leaf samples were boiled in a bleaching solution (ethanol:acetic acid:glycerol = 3:1:1) for 15 min.

### Statistical Analysis

Statistical analysis was performed using IBM SPSS Statistics version 25. Differences at *P* < 0.05 were considered significant.

## Results

### CgNLP1 Was a Type I NLP Candidate Effector

**Figure 1:**
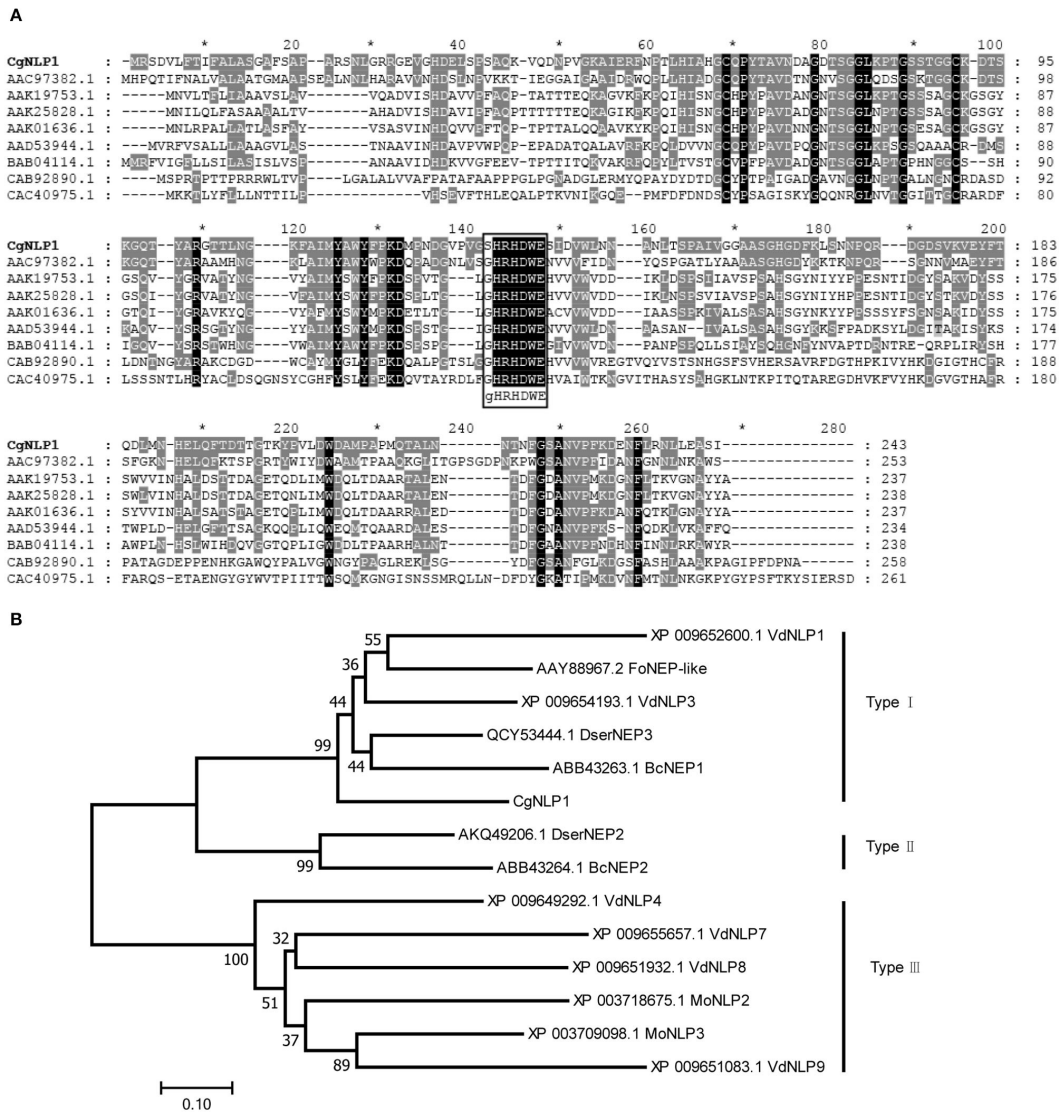
Multiple sequence alignment and phylogenetic analysis of CgNLP1. **(A)** Alignment of the necrosis- and ethylene-inducing protein 1 (Nep1)-like protein (NLP) from different microorganisms. Shading indicates regions of conservation in all (black), the same amino acid as CgNLP1 (gray) of sequences. NLPs used for alignment are from *Fusarium oxysporum* (AAC97382.1), *Pythium aphanidermatum* (AAD53944.1), *Phytophthora infestans* (AAK25828.1), *Phytophthora parasitica* (AAK19753.1), *Phytophthora sojae* (AAK01636.1), *Streptomyces coelicolor* A3(2) (CAB92890.1), *Alkalihalobacillus halodurans* C-125 (BAB04114.1), and *Vibrio pommerensis* (CAC40975.1). **(B)** Phylogenetic tree of CgNLP1 with different types of NLPs in fungi. VdNLP1 and VdNLP3 are from *Verticillium dahlia*, DserNEP3 is from *Diplodia seriata*, FoNEP-like is from *Fusarium oxysporum*, BcNEP1 and BcNEP2 are from *Botrytis cinerea*, DserNEP2 is from *Diplodia seriata*, PcNPP1 is from *Phytophthora cinnamomi*, MoNLP2 and MoNLP3 are from *Pyricularia oryzae*, and VdNLP4, VdNLP7, VdNLP8, and VdNLP9 are from *Verticillium dahlia*.

Based on the genome sequencing of *C. gloeosporioides* from *hevea brasiliensis*, the genes encoding extracellular secretory protein were predicted and one of them, named *CgNLP1* (ON402370), was amplified by RT-PCR. The open reading frame (ORF) of *CgNLP1* was 729 bp encoding a protein of 243 aa with two cysteine residues and a signal peptide (1-18aa) at its N-terminal (Supplementary Figure S1). The amino acid sequences of CgNLP1 were aligned with some NLP proteins identified in fungus, oomycete, and bacteria (Figure 1A). The alignment showed that CgNLP1 contained a typical NPP1 domain with a heptapeptide motif SHRHDWE and had the highest homology with NLP protein from *Verticillium dahliae*. The different types of NLP proteins identified in fungal species including CgNLP1 were used to generate a neighbor-joining tree (Figure 1B). Phylogenetic tree analysis revealed that CgNLP1 was clustered in the same branch as the other type I NLP proteins. These results suggested that *CgNLP1* encoded a type I NLP effector protein.

### CgNLP1 Contributed to the Virulence of *C. gloeosporioides* in the Rubber Tree

**Figure 2:**
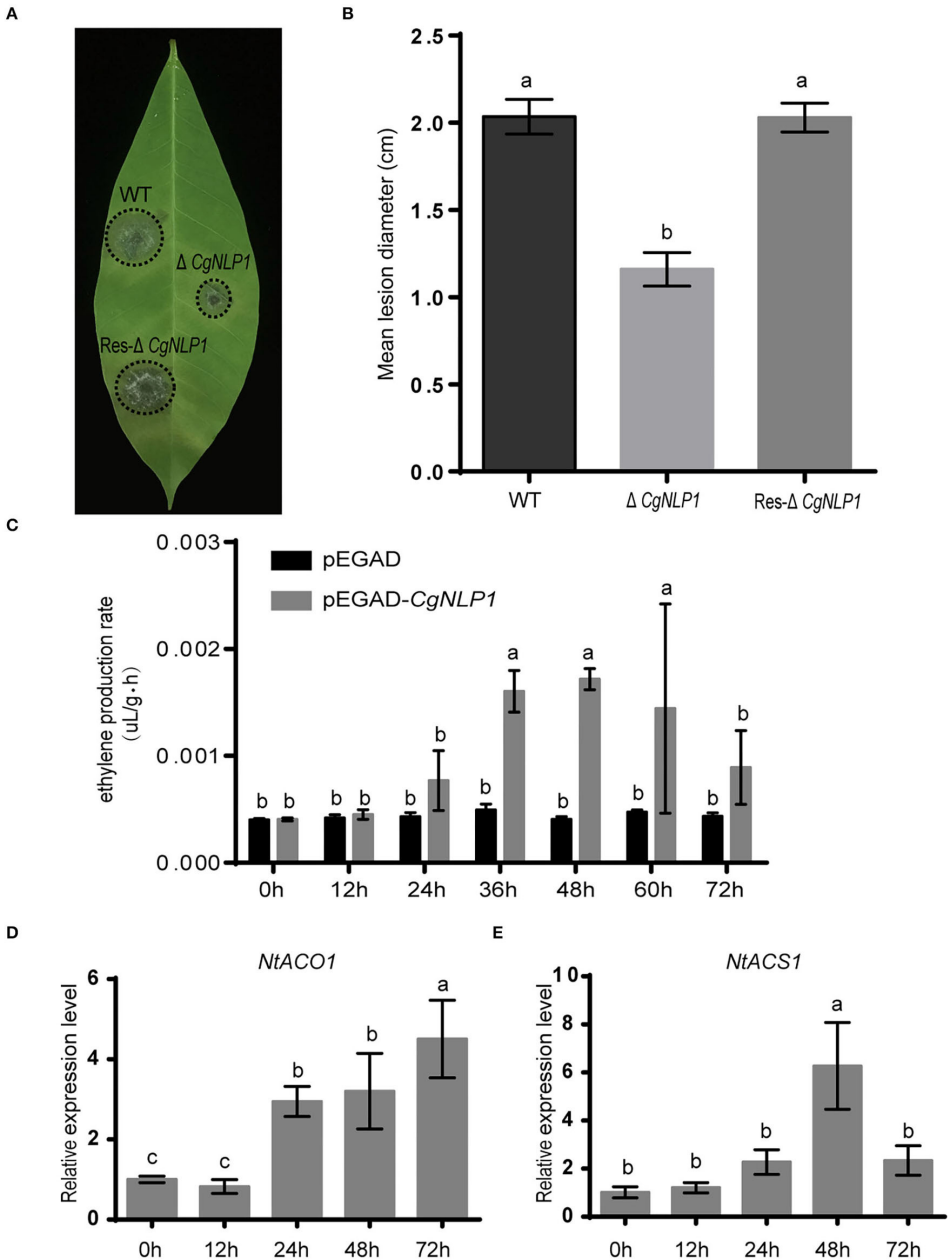
CgNLP1 contributed to the virulence of *Colletotrichum gloeosporioides* in the rubber tree and induced ethylene production in plants. **(A)** Disease symptoms on rubber tree leaves at 4 days of post-inoculated with conidia of Δ*CgNLP1*, Res-Δ*CgNLP1*, and wild type (WT). **(B)** Statistical analysis of lesion diameter after inoculation with WT, Δ*CgNLP1*, and Res-ΔCgNLP1. **(C)** The ethylene content in tobacco leaves expressing *CgNLP1*. pEGAD represents the tobacco leaves expressing empty vector pEGAD, and pEGAD-CgNLP1 represents the tobacco leaves expressing constructive vector pEGAD-CgNLP1. **(D)** The expression pattern of *NtACO1* (LOC107781126) in tobacco leaves expressing CgNLP1. **(E)** The expression pattern of *NtACS1* (LOC107831434) in tobacco leaves expressing CgNLP1 at different time points. Data are shown as the means ± standard deviation (SD) from three independent experiments, and columns with different letters indicate a significant difference (*P* < 0.05).

The *CgNLP1* knockout mutant (Δ*CgNLP1*) was obtained through gene homologous recombination technology, and its complementary strain (Res-Δ*CgNLP1*) was generated by introducing *CgNLP1* into Δ*CgNLP1* (Supplementary Figure S2). The detached leaf inoculation assay of Δ*CgNLP1* and Res-Δ*CgNLP1* on the rubber tree was performed to explore the contribution of *CgNLP1* to the pathogenicity of *C. gloeosporioides*. Results showed that typical necrotic lesions were observed in the leaves inoculated with wild type (WT), Δ*CgNLP1*, and Res-Δ*CgNLP1* (Figure 2A). At 4 dpi, the lesion size caused by Δ*CgNLP1* was significantly smaller than that caused by WT, and there was no significant difference in the lesion size caused by Res-Δ*CgNLP1* compared with the WT (Figure 2B). These results indicated that the loss of *CgNLP1* resulted in reduced pathogenicity of *C. gloeosporioides*, suggesting the contribution of *CgNLP1* to the virulence of *C. gloeosporioides* in the rubber tree.

### CgNLP1 Induced Ethylene Production but Not Cell Death

To determine the necrosis and ethylene-inducing ability of CgNLP1 in plants, tissue necrosis observation and the ethylene content determination were performed in the tobacco leaves transiently expressing *CgNLP1* by agroinfiltration. No obvious tissue necrosis was observed in the infiltration area of tobacco leaves within 3 days post infiltration, and Trypan blue staining results showed no obvious difference between the leaves infiltrated with *A. tumefaciens* GV3101 harboring *CgNLP1* gene and empty vector (Supplementary Figure S3). The ethylene content in the leaves expressing CgNLP1 was significantly higher than that in the leaves expressing empty vector (Figure 2C), and the expression levels of ACO and ACS, which are the two key enzymes of ethylene synthesis, were significantly higher in leaves expressing NLP than that in control (Figures 2D,E). These data suggested that CgNLP1 induced ethylene production in the plant but not necrosis and cell death.

### CgNLP1 Affected the Growth and Development of *C. gloeosporioides*

**Figure 3:**
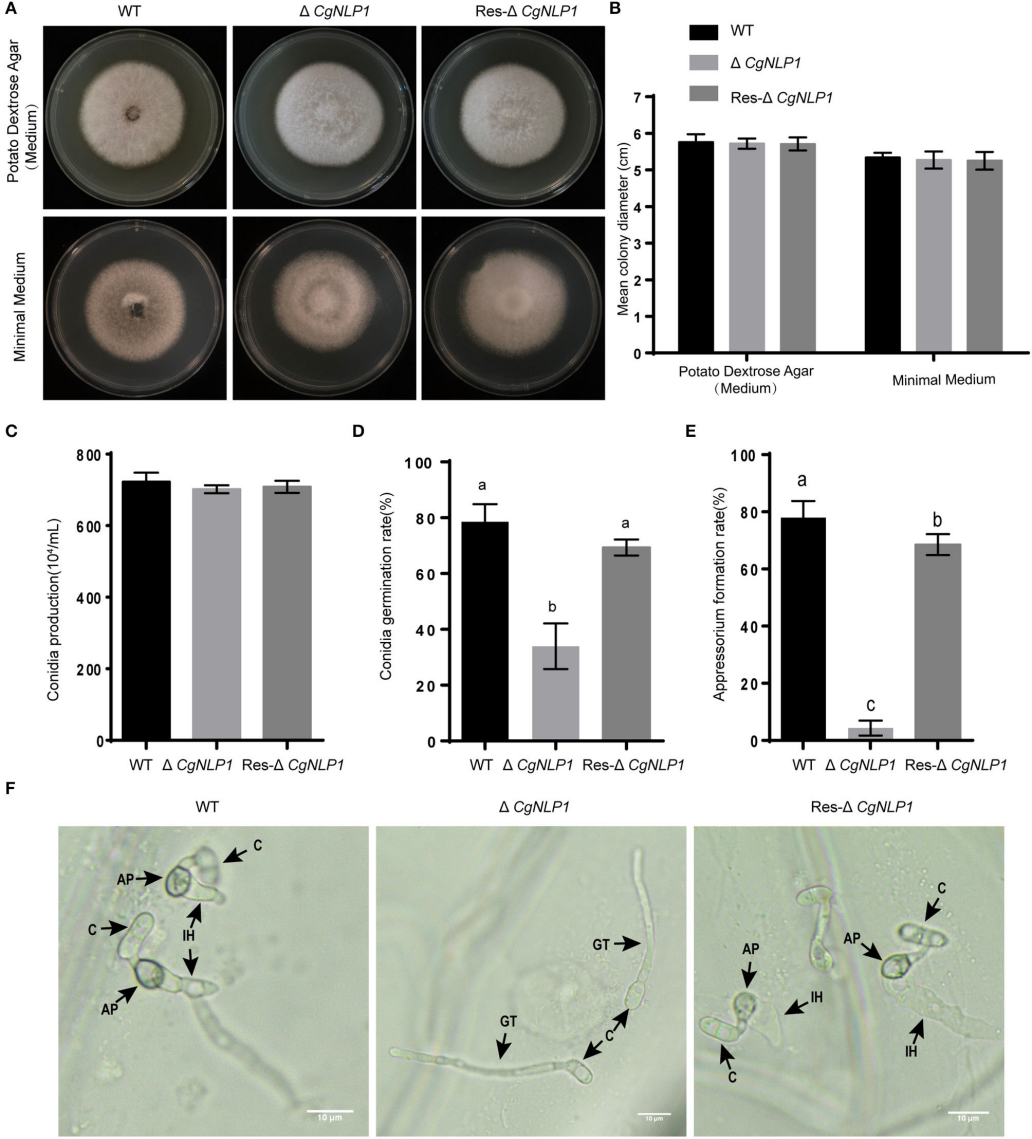
The radial growth, conidiation, and appressorium formation assays of CgNLP1 deletion mutant and CgNLP1 complementation mutant. **(A)** Hyphal blocks from Cg-WT, ΔCgNLP1, and Res-ΔCgNLP1 were inoculated on potato dextrose agar (PDA) medium and minimal medium for 6 days at 28°C, and this experiment was repeated three times. **(B)** Colony diameters at 6 days of post-inoculation. **(C)** The number of conidia developed by Cg-WT, ΔCgNLP1, and Res-ΔCgNLP1. **(D)** Conidial germination rates of Cg-WT, ΔCgNLP1, and Res-ΔCgNLP1 at 4 h time intervals. **(E)** Appressorium formation rates of Cg-WT, ΔCgNLP1, and Res-ΔCgNLP1 at 12 h time intervals. Error bars indicate SD, and columns with different letters indicate a significant difference (*P* < 0.05). **(F)** Equal volumes (30 μl) of conidial suspensions (2.5 × 10^5^ conidia/ml) from Cg-WT, ΔCgNLP1, and Res-ΔCgNLP1 were inoculated on the onion epidermal cells. C, AP, IH, and GT indicate the conidia, appressorium, infection hyphae, and germ tube, separately. This experiment was repeated three times. Bars = 10 μm.

To investigate the roles of CgNLP1 on growth and development, various growth characteristics were assessed. Radial growth and conidiation of Δ*CgNLP1* were similar to that of WT and Res-Δ*CgNLP1* (Figures 3A–C). However, less than 30% of the conidia germinated after 4 h of post-incubation in Δ*CgNLP1*, compared with approximately 80% in WT and Res-Δ*CgNLP1* (Figure 3D); only less than 10% of conidia formed appressoria after 12 h of post-incubation in ΔCgNLP1, compared with approximately 80% in WT and Res-Δ*CgNLP1* (Figure 3E). Invasive growth assay showed that WT and Res-Δ*CgNLP1* formed primary hypha in onion epidermis, but Δ*CgNLP1* failed to form primary hypha (Figure 3F). These results indicated that CgNLP1 was involved in conidial germination, appressorium formation, and invasive growth but not radial growth and conidiation.

### Ectopic Expression of *CgNLP1* Enhanced Plant Disease Resistance

**Figure 4:**
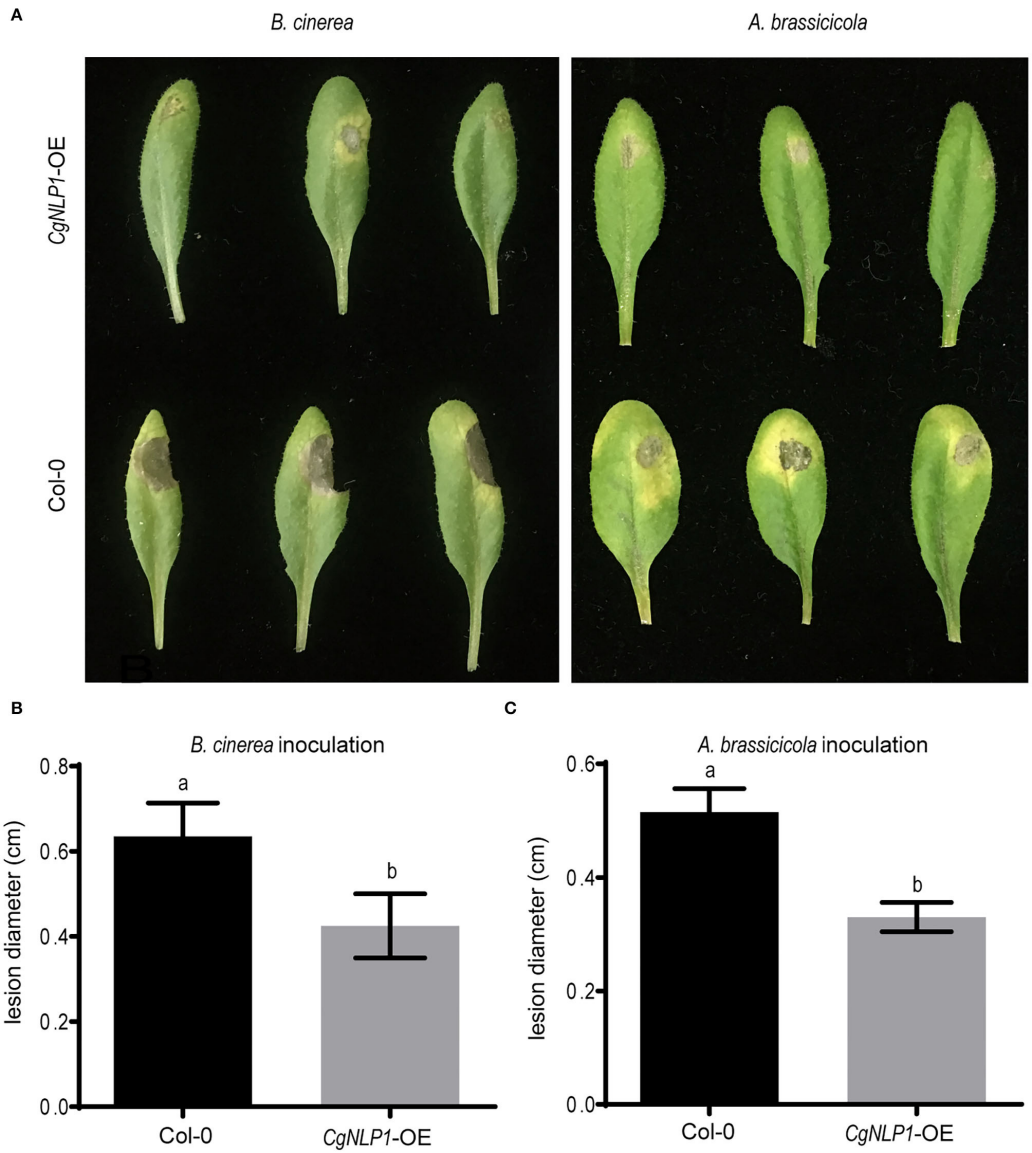
Overexpression of *CgNLP1* in *Arabidopsis* enhanced the resistance to *B. cinerea* and *Alternaria brassicicola*. **(A)** Disease symptoms on *Arabidopsis* lines overexpressing *CgNLP1* (*CgNLP1*-OE) and WT (Col-0) at 3 days of post-inoculated with *B. cinerea* and *A. brassicicola*, respectively. **(B,C)** Statistical analysis of lesion diameter on CgNLP1-OE and *Col-0* after inoculation with *B. cinerea* and *A. brassicicola*. Data are shown as the means ± SD from three independent experiments, and columns with different letters indicate a significant difference (*P* < 0.05).

To investigate the roles of *CgNLP1* on plant disease resistance, *CgNLP1* transgenic *Arabidopsis* plants driven by the estradiol-induced promoter (*CgNLP1-OE*) were generated (Supplementary Figure S4). Disease assay showed that although typical necrotic lesions appeared on both Col-0 and *CgNLP1-OE* at 4 days of post-inoculation with *B. cinerea* and *A. brassicicola*, respectively (Figure 4A), the lesion size on *CgNLP1-OE* was significantly smaller than that on Col-0 (Figures 4B,C). These results indicated that the ectopic expression of *CgNLP1* in *Arabidopsis* significantly enhanced resistance to *B. cinerea* and *A. brassicicola*, suggesting the function of *CgNLP1* as an elicitor.

### CgNLP1 Targeted Rubber Tree TF HbMYB8-Like

To elucidate the pathogenic mechanism of *C. gloeosporioides* on the rubber tree, the targets of CgNLP1 were identified from a cDNA library of rubber tree leaves by yeast two-hybrid using the full-length CgNLP1 as bait. After initial screening, 25 positive clones were sequenced, and five of them were chosen for candidates. After verification, it is found that four of them had strong self-activation, and only one of them, named HbMYB8-like (LOC110651555), which is self-activation, could be inhibited by adding an appropriate concentration of Aba. The *HbMYB8-like* gene was amplified by RT-PCR and verified by sequencing. The result showed that the full-length cDNA of the *HbMYB8-like* gene was 1,183 bp with a 903 bp ORF encoding 300 amino acids. The HbMYB8-like protein contained two SANT domains, which were typical features of MYB TFs. The results of multiple sequence alignment (Supplementary Figure S5) and phylogenetic tree (Supplementary Figure S6) showed that HbMYB8-like protein had typical adjacent repeats R2R3 and was clustered clusters with R2R3-MYB type MYB TFs from other plants, suggesting that HbMYB8-like belonged to the R2R3 type MYB TFs.

**Figure 5:**
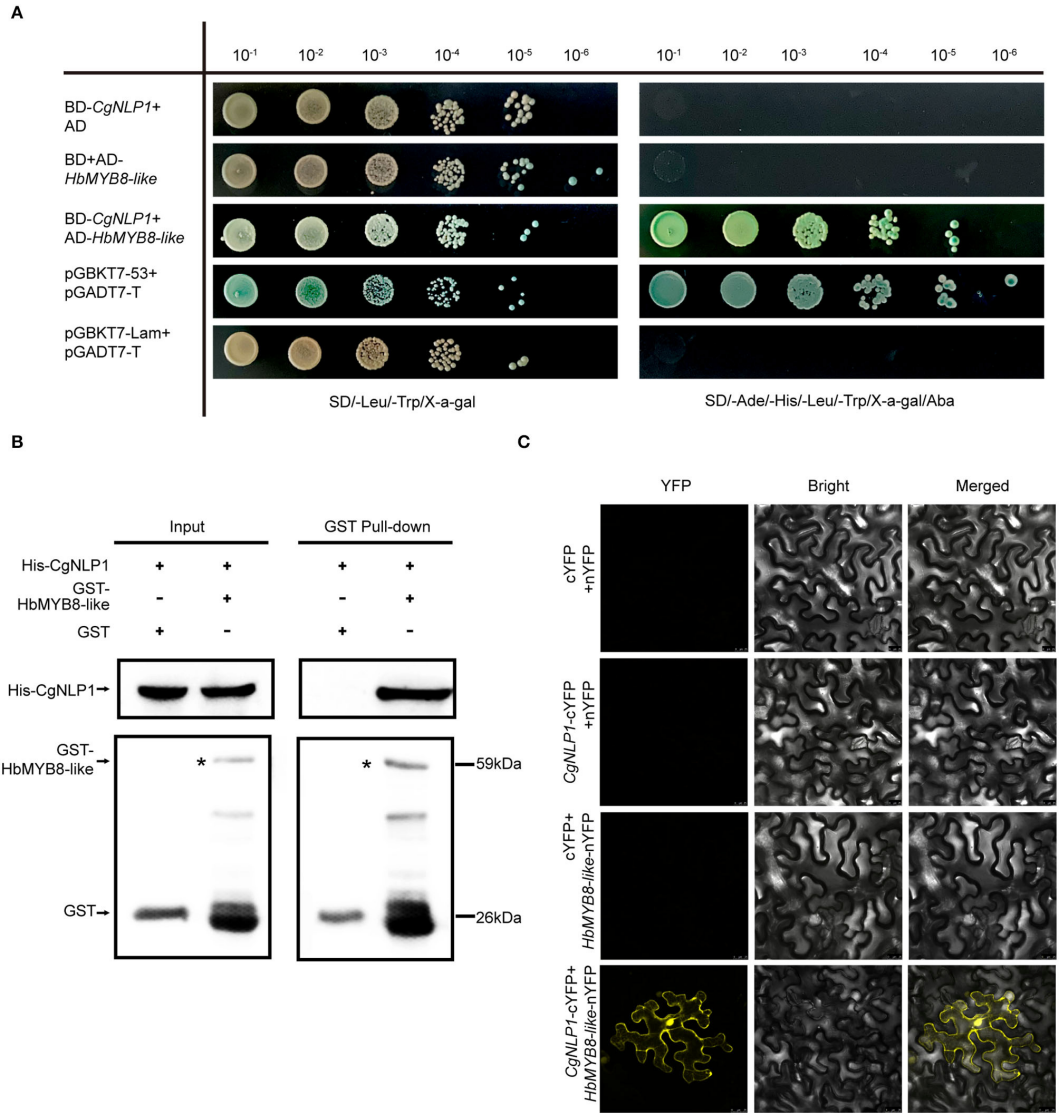
Screening and verification of the interaction between CgNLP1 and HbMYB8-like. **(A)** Verification of the interaction between CgNLP1 and HbMYB8-like by yeast two-hybrid assay. AD indicates pGADT7, BD indicates pGBKT7, pGADT7-T + pGBKT7-53 indicates a positive control, and pGBKT7-Lam+pGADT7-T indicates a negative control. SD/-Leu/-Trp/X-α-gal indicates the medium with X-α-gal, but lacking Leu and Trp. SD/-Ade/-His/-Leu/-Trp/Xa-gal/Aba indicates the medium with X-α-gal and Aba, but lacking Ade, His, Leu, and Trp. 10^−1^, 10^−2^, 10^−3^, 10^−4^, 10^−5^, and 10^−6^, respectively, refer to the yeast suspension with the initial optical density (OD) value of 2.0 being diluted in a 10-fold gradient. **(B)** Verification of the interaction between CgNLP1 and HbMYB8-like by GST pull-down assay. His and GST stand for pCold^TM^ TF and pGEX, respectively. Arrows indicate GST- and His-tagged proteins. **(C)** Verification of the interaction between CgNLP1 and HbMYB8-like by bimolecular fluorescence complementation (BiFC) assay. cYFP + nYFP, *CgNLP1*-cYFP + nYFP, and cYFP + *HbMYB8-like*-nYFP were used as a negative control. Scale bar = 25 μm.

The interaction of CgNLP1 and full-length HbMYB8-like was preliminarily verified through yeast two-hybrid (Figure 5A). Then, pull-down and BiFC assays were performed to further verify the interaction between CgNLP1 and HbMYB8-like *in vitro*. In pull-down assay, His-tagged CgNLP1 and GST-tagged HbMYB8-like proteins were expressed in *E. coli* BL21 (DE3) respectively. The supernatant containing GST-tagged HbMYB8-like proteins were precipitated using anti-GST beads after co-incubation with supernatants containing His-tagged CgNLP1 and His protein only, respectively, and the precipitates were detected using anti-His and anti-GST antibodies. It was found that His-tagged CgNLP1could be pulled down by GST-tagged HbMYB8-like protein (Figure 5B). In the BiFC assay, CgNLP1 was translationally fused with the C-terminal portion of YFP (CgNLP1-cYFP), and HbMYB8-like was fused with the N-terminal portion of YFP (HbMYB8-like -nYFP). CgNLP1-cYFP and HbMYB8-like-nYFP were introduced into *A. tumefaciens* and co-infiltrated into *N. benthamiana* leaves. Microscopic examination revealed YFP fluorescence only when the two constructs were co-expressed. Leaves from plants infiltrated with either of the constructs alone or in combination with the empty vector showed no fluorescence (Figure 5C). These results demonstrated that CgNLP1 interacted with HbMYB8-like.

### HbMYB8-Like Protein Localized on Nucleus and Induced Cell Death

**Figure 6:**
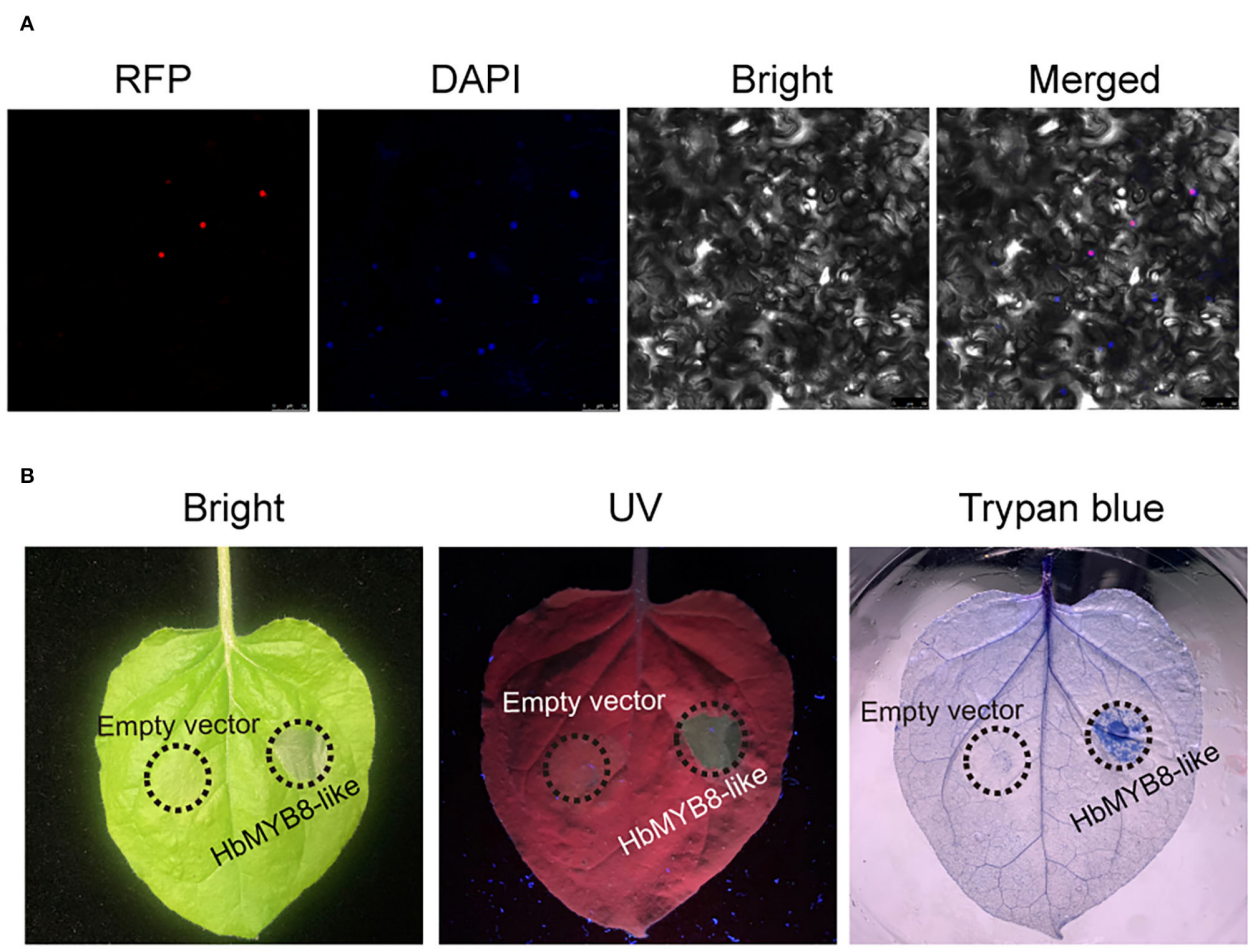
HbMYB8-like localized on the nucleus and induced necrotic cell death. **(A)** Subcellular localization of HbMYB8-like protein in tobacco leaves. **(B)** Cellular necrosis induced by HbMYB8-like in tobacco leaves. Leaves were photographed under UV illumination (right) and normal light (left).

The HbMYB8-like-RFP fusion protein was transiently expressed in *N. benthamiana* by agroinfiltration to determine the subcellular localization and explore the function of defense response. In the leaf tissues expressing only RFP, red fluorescence was observed throughout the entire cells. However, in the tissues expressing HbMYB8-like-RFP fusion proteins, red fluorescence was observed only on the nucleus (Figure 6A). After 2 days of post-infiltration with *A. tumefaciens* harboring the *HbMYB8-like-RFP* gene, significant necrosis was observed in the infiltration area of tobacco leaf, while no necrosis was observed on the leaf infiltrated with *A. tumefaciens* harboring only RFP gene. Meanwhile, cell death was observed in the infiltrated with *A. tumefaciens* GV3101 harboring the *HbMYB8-like-RFP* gene (Figure 6B), suggesting that HbMYB8-like could induce cell death in tobacco leaves.

### *HbMYB8-Like* Responded to Fungal Phytopathogens and Phytohormones in Rubber Tree

**Figure 7:**
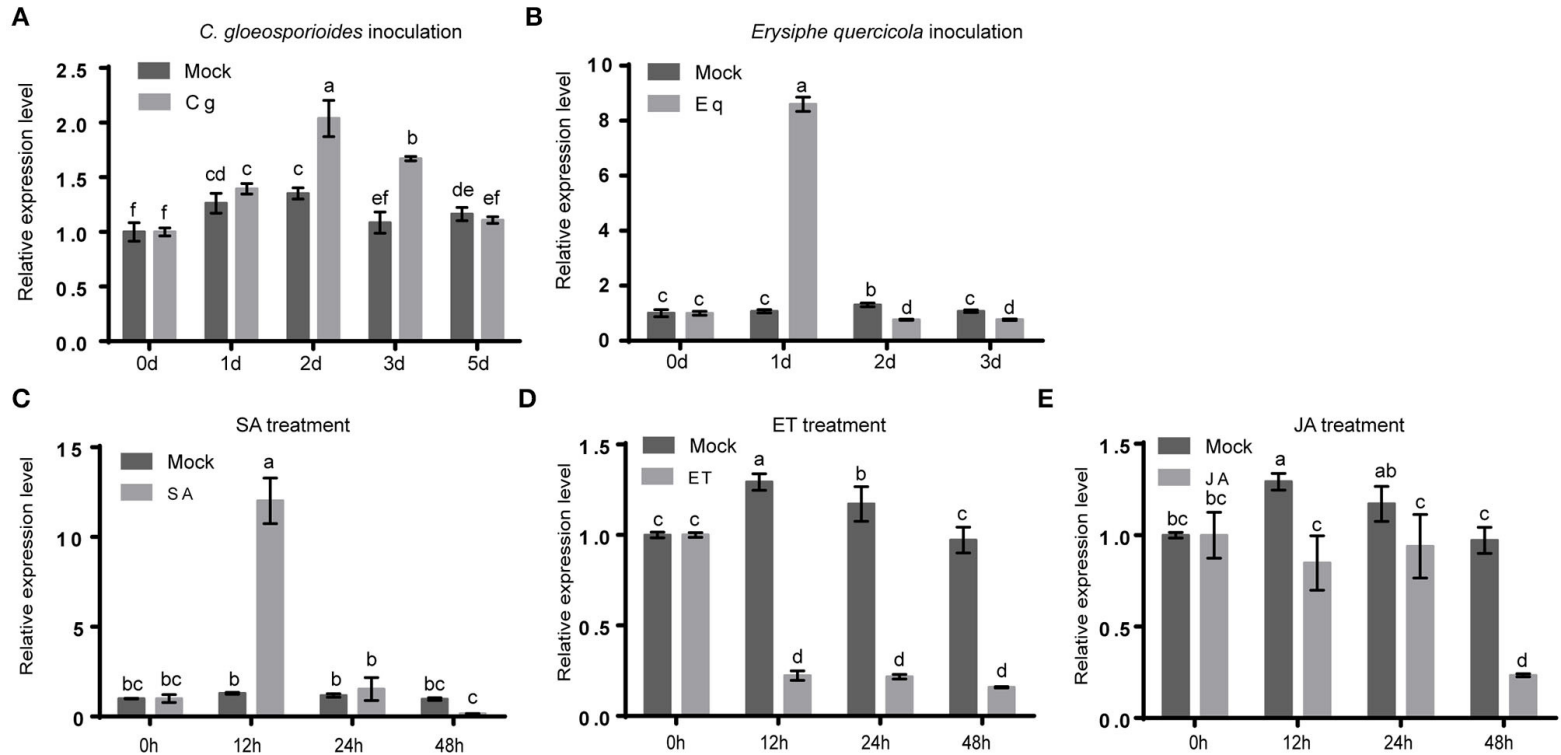
Expression profiles of *HbMYB8-like* in rubber tree leaves with phathomycete inoculation and phytohormones treatments. Data are shown as the means ± SD from three independent experiments, and columns with different letters indicate a significant difference (*P* < 0.05). **(A)** The expression profile of HbMYB8-like to C. gloeosporioides. **(B)** The expression profile of HbMYB8-like to *E. quercicola*. **(C)** The expression profile of HbMYB8-like to SA. **(D)** The expression profile of HbMyb8-like to ET. **(E)** The expression profile of HbMyb8-like to JA.

To explore the possible roles of *HbMYB8-like* in disease resistance of rubber tree, the expression profiles of *HbMYB8-like* were investigated in responding to *C. gloeosporioides* and *Erysiphe quercicola* which caused rubber tree anthracnose and powdery mildew, respectively. In the rubber tree leaves inoculated with *C. gloeosporioides* and *E. quercicola*, the expression of *HbMYB8-like* was upregulated significantly at 24 h of post-inoculation (hpi) (Figures 7A,B). In addition, the expression profiles of *HbMYB8-like* responding to different phytohormones were also investigated. The results showed that the expression of *HbMYB8-like* was significantly induced more than 8 times at 12 h after SA treatment and was significantly downregulated by JA and ET treatments (Figures 7C–E). These results suggested that *HbMYB8-like* was involved in the resistance to fungal phytopathogens through SA-, JA-, and ET-mediated signaling in the rubber tree.

### CgNLP1 Disrupted the Nuclear Accumulation of HbMYB8-Like and Inhibited HbMYB8-Like Induced Cell Death

**Figure 8:**
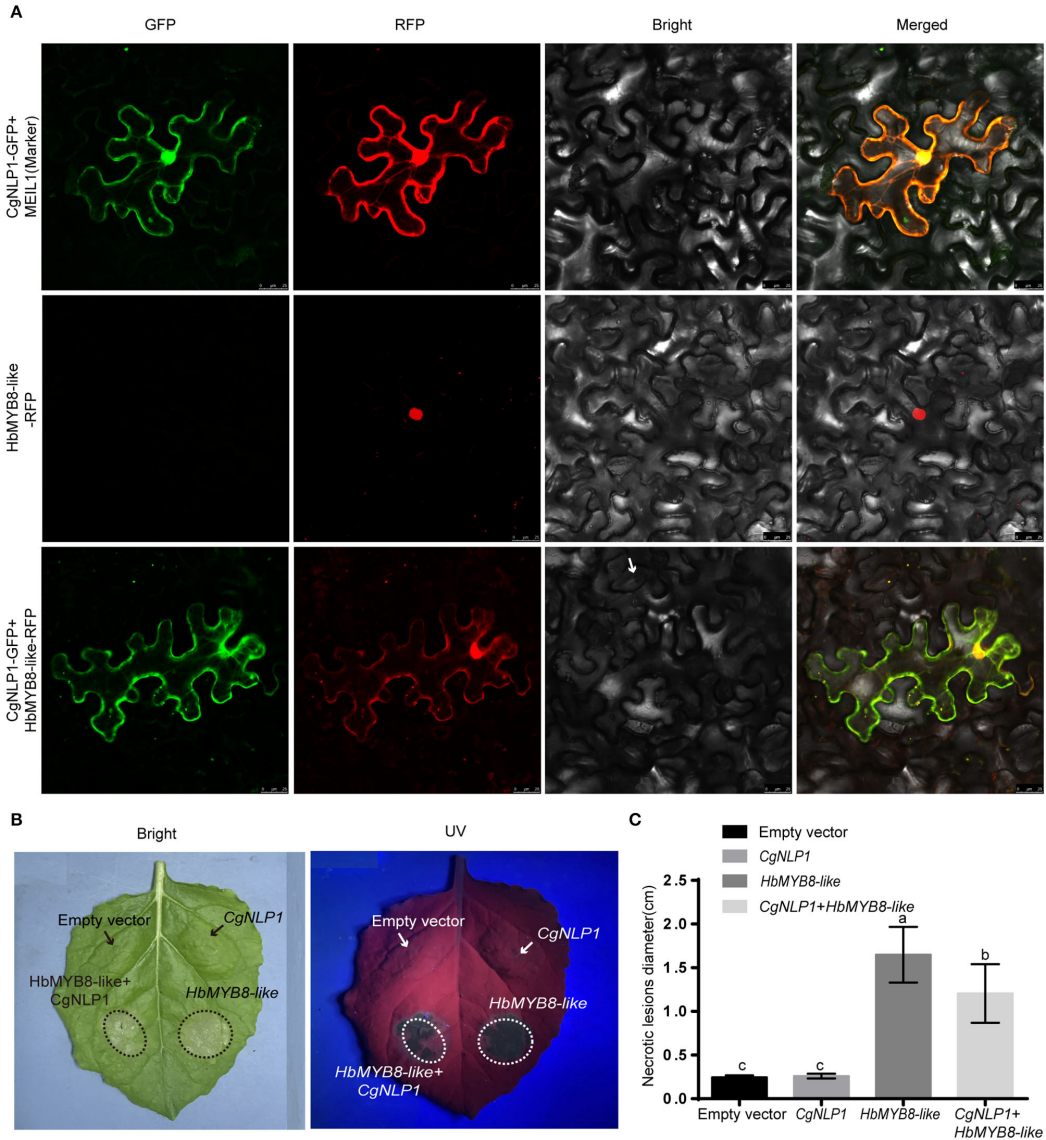
CgNLP1 repressed nuclear accumulation of HbMYB8-like and inhibited necrosis induced by HbMYB8-like. **(A)** CgNLP1 repressed nuclear accumulation of HbMYB8-like. Scale bar = 25 μm. **(B)** CgNLP1 inhibited necrosis induced by HbMYB8-like. **(C)** Statistical analysis of necrosis diameter in tobacco leaves. Data are shown as the means ± SD from three independent experiments, and columns with different letters indicate a significant difference (*P* < 0.05).

We had demonstrated that HbMYB8-like localized on the nucleus as a TF and induced necrosis in tobacco tissues (Figure 6). When CgNLP1-GFP fusion and nucleocytoplasmic marker (MIEL1-RFP) were transiently co-expressed in *N. benthamiana* leaves, the CgNLP1-GFP completely overlaps with the MIEL1-RFP, indicating that CgNLP1 was localized on the nucleus and cell membrane. Compared with the uniform distribution of HbMYB8-like-RFP in the nuclei, when HbMYB8-like-RFP and CgNLP1-GFP were transiently co-expressed in *N. benthamiana* leaves, less HbMYB8-like-RFP was observed in nuclei, and instead, a substantial amount of HbMYB8-like was localized on the cell membrane (Figure 8A). In addition, the necrosis size caused by co-expression of HbMYB8-like-RFP with CgNLP1-GFP was significantly smaller than that caused by expression of HbMYB8-like-RFP only (Figures 8B,C). These data suggested that CgNLP1 could interfere in the nuclear accumulation of HbMYB8-like and inhibit HbMYB8-like induced necrosis and cell death.

### CgNLP1 Inhibited SA Signaling Mediated by HbMYB8-Like in Tobacco Leaves

**Figure 9:**
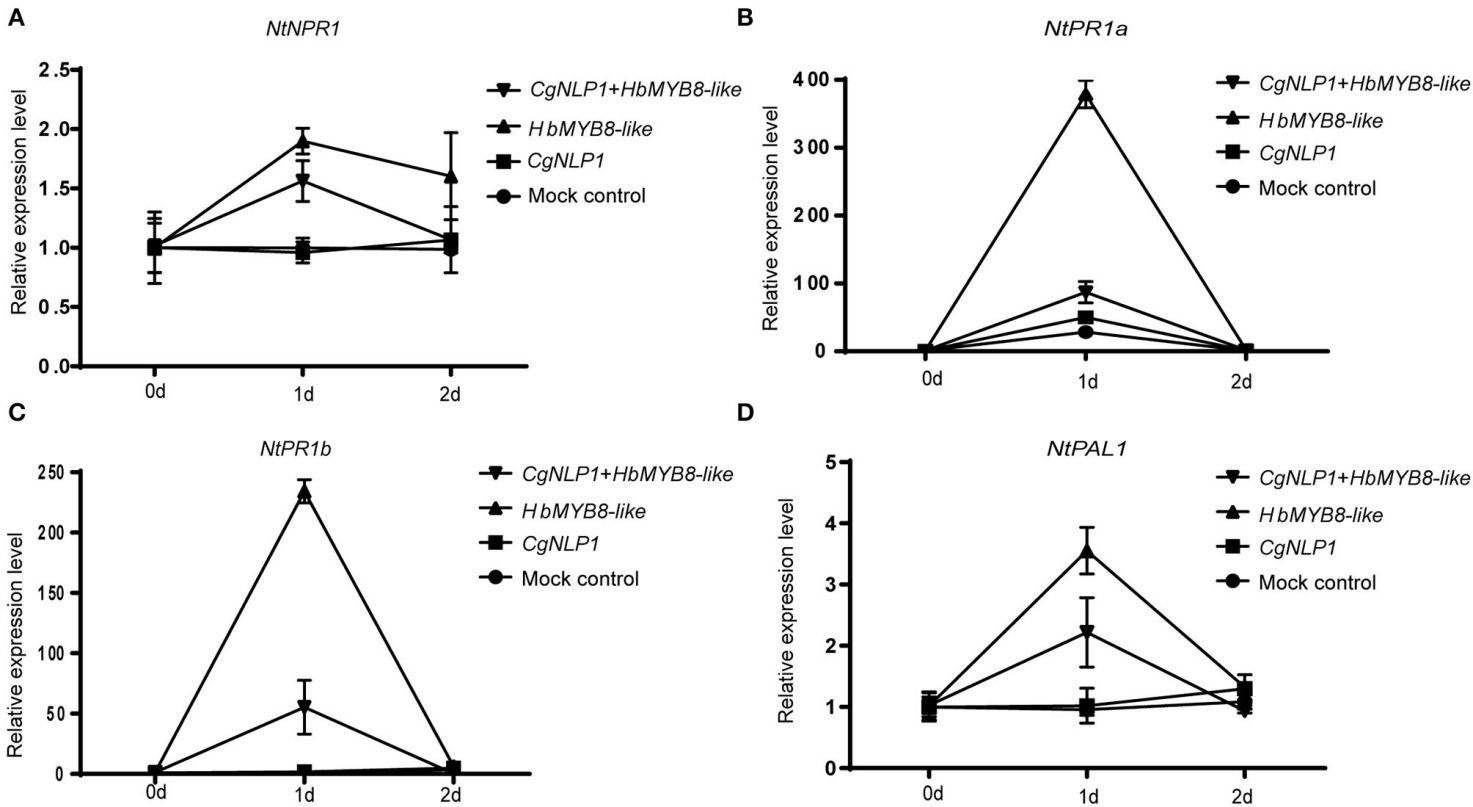
**(A–D)** Expression patterns of genes related to the salicylic acid (SA) signaling pathway in the tobacco leaves expressing HbMYB8-like and co-expressing HbMYB8-like and CgNLP1. Data are shown as the means ± SD from three independent experiments.

Since CgNLP1 could induce ethylene production in plants and HbMYB8 was upregulated in response to exogenous SA (Figures 2C, 7), the expression patterns of genes related to SA signaling pathways such as *NtPR1a, NtPR1b, NtNPR1*, and *NtPAL1* were examined in the tobacco leaves expressing HbMYB8-like and co-expressing HbMYB8-like and CgNLP1, respectively. As shown in Figure 9, the expression of *NtPR1a, NtPR1b, NtNPR1*, and *NtPAL1* had been enhanced significantly in the tobacco leaves expressing HbMYB8-like for 24 h, compared with that in the tobacco leaves expressing CgNLP1 and empty vector (mock control) but reduced significantly in the tobacco leaves co-expressing HbMYB8-like and CgNLP1. These results indicated that CgNLP1 inhibited SA signaling, which was mediated by HbMYB8-like.

## Discussion

In the 30 years since the first NLP protein was discovered from the culture filtrate of *Fusarium oxysporum* f.sp. *erythroxyli* (Bailey, 1995), a large number of NLPs had been identified based on a prominent feature of the NPP1 domain with a conserved heptapeptide motif GHRHDWE (Oome and Van den Ackerveken, 2014). As mentioned in the “Introduction” section, NLP could be divided into three types, which differed especially in the number of cysteines. Type I NLPs contained two cysteines forming a conserved disulfide bond, and type II had four cysteines forming two conserved disulfide bonds and an additional putative calcium-binding domain (Oome et al., 2014). In our study, CgNLP1, an NLP protein identified from *C. gloeosporioides*, contained an NPP1 domain with two cysteine residues (Figure 1A), which conformed to the structural characteristics of type I NLPs. So, we identified it as type I NLP protein, and it was also supported by phylogenetic analysis (Figure 1B).

As the name suggests, most NLPs have the ability to induce necrosis and ethylene production in eudicot plants (Bailey, 1995; Gijzen and Nürnberger, 2006; Chen et al., 2018). In our case, CgNLP1 could promote ethylene synthesis and accumulation in plant tissues (Figure 2C) but not necrosis and cell death (Supplementary Figure S3). Signal peptides at N-terminal, two or four cysteine residues, and heptapeptide motif GHRHDWE were directly related to the necrosis-inducing ability of NLPs (Zaparoli et al., 2011; Lenarči et al., 2017). When one cysteine was replaced, the NLP protein from *Phytophthora parasitica*, NPP1, lost the necrosis-inducing ability (Fellbrich et al., 2002). Both *P. parasitica* NLPPp and *P. sojae* NLPPs, without signal peptide, did not show cytotoxicity when transiently expressed in sugar beet (Qutob et al., 2006). In conserved heptapeptide motif GHRHDWE of NLP from *Moniliophthora perniciosa*, the substitution of either at His2 (H) or Asp4 **(D)** with alanine substantially abrogated its necrosis-inducing activity (Ottmann et al., 2009; Zaparoli et al., 2011). However, HaNLP3, an NLP protein from *H. arabidopsidis*, did not induce necrosis on plant cells despite having a conserved heptapeptide motif (Ottmann et al., 2009; Cabral et al., 2012). In our case, CgNLP1 contained a signal peptide, two cysteine residues, and an SHRHDWE motif with His2 (H) and Asp4 **(D)**, which was different from the typical heptapeptide motif GHRHDWE at the first amino acid site. Thus, signal peptide, cysteine residue number, and conserved SHRHDWE motif do not appear to be necessary for CgNLP1 to induce necrosis and cell death. Further analysis is needed to determine whether the first amino acid “S” in the heptapeptide motif has an effect on the necrosis-inducing ability of CgNLP1.

Several studies showed that NLPs from different pathogens play distinct roles in pathogen virulence (Xiang et al., 2022). Both NLP1 and NLP2 from *Verticillium dahlia* induced cell death and were required for virulence, but only NLPs affected vegetative growth and conidiospore production (Santhanam et al., 2013). The deletion of NLP in *Erwinia carotovora* attenuated the virulence on both rubbers and stems of potatoes (Pemberton et al., 2005). Three NLPs from *Phytophthora capsica*, namely, PcNLP2, PcNLP6, and PcNLP14, contributed to strong virulence during infection in both pepper and tobacco plants (Feng et al., 2014). The introduction of Nep1 from *F. oxysporum* into a hypovirulent *Colletotrichum coccodes* strain dramatically increased its virulence and expanded its host spectrum (Amsellem et al., 2002). However, the deletion of NLPs in *Botrytis elliptica, Mycosphaerella graminicola*, and *Magnaporthe oryzae* did not reduce the virulence of these pathogens on each host (Staats et al., 2007; Motteram et al., 2009; Zhou et al., 2012; Fang et al., 2017). Our results showed that the disruption of *CgNLP1* not only affected conidial germination, appressorium formation, and invasive growth but also impaired the pathogenicity of *C. gloeosporioides* to the rubber tree (Figures 2, 3), indicating that CgNLP1 played multiple roles in the virulence of C. *gloeosporioides*.

Increasing observations showed that NLPs could induce plant innate immune responses, resulting in enhanced disease resistance (Seidl and Ackerveken, 2019). For example, ectopic expression of HaNLP3 in *Arabidopsis* enhanced the resistance to downy mildew (Oome and Van den Ackerveken, 2014). In our study, ectopic expression of CgNLP1 in *Arabidopsis* enhanced the resistance to *B. cinerea* and *A. brassicicola* (Figure 4), indicating that *CgNLP1* performed as an elicitor. In addition, hormone signaling crosstalk played major roles in plant defense against pathogens (Derksena et al., 2013). SA, JA, and ET play critical roles in the regulation of signaling networks of basal resistance against multiple pathogens (Pieterse et al., 2012). SA signaling positively induces plant defense against biotrophic pathogens, whereas the JA/ET pathways are required for resistance predominantly against necrotrophic pathogens and herbivorous insects (Yang et al., 2015). CgNLP1 could not only enhance the disease resistance of *Arabidopsis* to necrotrophic fungi *B. cinerea* and *A. brassicicola* (Figure 4) but also promote ethylene synthesis and accumulation in plant tissues (Figures 2C–E), indicating that the disease resistance triggered by CgNLP1 to necrotrophic pathogens may be related to ethylene accumulation and enhanced ethylene-mediated signaling pathway.

Fungal effectors were usually secreted and delivered into host plants as pathogenic factors to shield the fungus and suppress the host immune response, or manipulate host cell physiology by targeting plant defense components, signaling, and metabolic pathways to promote host plant colonization (Lo Presti et al., 2015). Plant TFs played roles in diverse biological processes including defense responses to pathogens (Seo and Choi, 2015). Several studies had demonstrated that TFs were targets of effectors in plants. A bacterial effector AvrRps4 interacted with WRKY TFs (Sarris et al., 2015). A *P. infestans* effector Pi03192 prevented the translocation of NAC TFs from the endoplasmic reticulum to the nucleus (Hazel et al., 2013). PpEC23 from *Phakopsora pachyrhizi* targeted TF GmSPL12l to suppress host defense response (Qi et al., 2019). Stripe rust effector PstGSRE1 disrupts nuclear localization of TF TaLOL2 to defeat ROS-induced defense in wheat (Qi et al., 2012). In our study, CgNLP1 physically interacted with an R2R3 type MYB TF HbMYB8-like in the rubber tree, which was localized to the nucleus (Figure 5A), and the expression of CgNLP1 in tobacco leaves induced partial re-localization of HbMYB8-like from the nucleus to the plasma membrane (Figure 8A). However, the molecular mechanism by which CgNLP1 disrupts the localization of HbMYB8-like remains unknown.

In our study, HbMYB8-like could induce typical necrosis and cell death in tobacco leaves (Figure 6B), which was suppressed by CgNPL1 (Figures 8B,C). Moreover, *HbMYB8-like* could respond to phytohormones, especially SA, which could upregulate the expression level of *HbMYB8-like* more than 10 times (Figure 7C). SA is considered to play an important role in the regulation of programmed cell death or hypersensitive reaction in plants (Radojičić et al., 2018). So, we measured the effects of HbMYB8-like on SA signaling in tobacco leaves. The results showed that HbMYB8-like could enhance the expression of *NtPR1a, NtPR1b, NtNPR1*, and *NtPAL1*, which were involved in the SA signaling pathway, and the expression of these genes was inhibited significantly when co-expressing CgNPL1 and HbMYB8-like (Figure 9). These results strongly suggested that the cell death induced by HbMYB8-like was a form of defense, hypersensitive response (HR), and that the pathogenicity of CgNLP1 was achieved through inhibition of HR induced by HbMYB8-like.

In summary, we identified an important *C. gloeosporioides* effector CgNLP1 that played roles as a virulence factor to rubber tree and as an elicitor of plant defense responses. Moreover, CgNLP1 interfered with nuclear accumulation of TF HbMYB8-like and inhibited the HR-induced byHbMYB8-like. The inhibition of CgNLP1 on the defense response mediated by HbMYB8-like revealed a novel pathogenic strategy of *C. gloeosporioides*.

## Data Availability Statement

The original contributions presented in the study are included in the article/Supplementary Material, further inquiries can be directed to the corresponding author.

## Author Contributions

GY carried out most of the experiments and analyzed the data. JY helped in growing and harvesting the *Arabidopsis* plants. WW cloned the CgNLP1 gene. QW completed the microscope observation. QZ did the vector construction. GY, BA, and HL wrote the manuscript. CH revised the manuscript. All authors read and approved the final manuscript.

## Funding

This study was supported by grants from the Hainan Natural Science Foundation (Project No. 319QN167) and the National Natural Science Foundation of China (Grant Nos. 31860478 and 32060591).

## Conflict of Interest

The authors declare that the research was conducted in the absence of any commercial or financial relationships that could be construed as a potential conflict of interest.

## Publisher's Note

All claims expressed in this article are solely those of the authors and do not necessarily represent those of their affiliated organizations, or those of the publisher, the editors and the reviewers. Any product that may be evaluated in this article, or claim that may be made by its manufacturer, is not guaranteed or endorsed by the publisher.

## Supplementary Material

Supplementary Figure S1 Nucleotide sequence and deduced amino acid sequence of *CgNLP1*. Shading indicates the amino acid sequences of the signal peptide.

Supplementary Figure S2 Generation and molecular confirmation of *CgNLP1* deletion mutant (Δ*CgNLP1*) and complementation mutant (Res-ΔCg*NLP1*). **(A)** The diagram of the *CgNLP1* knockout vector. **(B)** Diagnostic PCR analysis for deletion of *CgNLP1* and integration of *CgNLP1* into the genome of *C. gloeosporioides*. **(C)** The expression level of *CgNLP1* in wild type (WT), Δ*CgNLP1*, and Res-Δ*CgNLP1* by quantitative RT-PCR.

Supplementary Figure S3 Trypan blue transient of tobacco leaves expressing *CgNLP1*. pEGAD indicates the tissue expressing empty vector pEGAD-eGFP, and pEGAD-CgNLP1 indicates the tissue expressing recombinant vector pEGAD-*CgNLP1*-eGFP.

Supplementary Figure S4 Identification of *CgNLP1* transgenic *Arabidopsis* lines by Western blot. Col-0 indicates *Arabidopsis* Columbia-0, and 1-6 indicates different CgNLP1-OE transgenic lines under the Col-0 background.

Supplementary Figure S5 Alignment of HbMYB8-like and homologs from different plants. Shading indicated regions of conservation in all (black), the same amino acid as HbMYB8-like (gray) sequences. The protein sequences used for alignment are as follows: *Arabidopsis thaliana* AtMYB6 (EFH48703.1), *Arabidopsis thaliana* AtMYB8 (Q9SDS8.1), *Oryza sativa* OsMYB30 (Q6K1S6.1), *Gossypium hirsutum* GhMYB1 (NP_001313761.1), and *Triticum aestivum* TaRIM1 (AMP18876.1).

Supplementary Figure S6 Phylogenetic tree of HbMYB8-like with different types of MYB proteins in plants.

Supplementary Table S1 The primers used for PCR amplification quantitative and RT-PCR.
