# Supplementary figures and images for: The Effector Protein CgNLP1 of Colletotrichum gloeosporioides Affects Invasion and Disrupts Nuclear Localization of Necrosis-Induced Transcription Factor HbMYB8-Like to Suppress Plant Defense Signaling
Source: Front Microbiol. 2022 Jun 13;13:911479. doi: 10.3389/fmicb.2022.911479 (PMC9234567; doi:10.3389/fmicb.2022.911479)

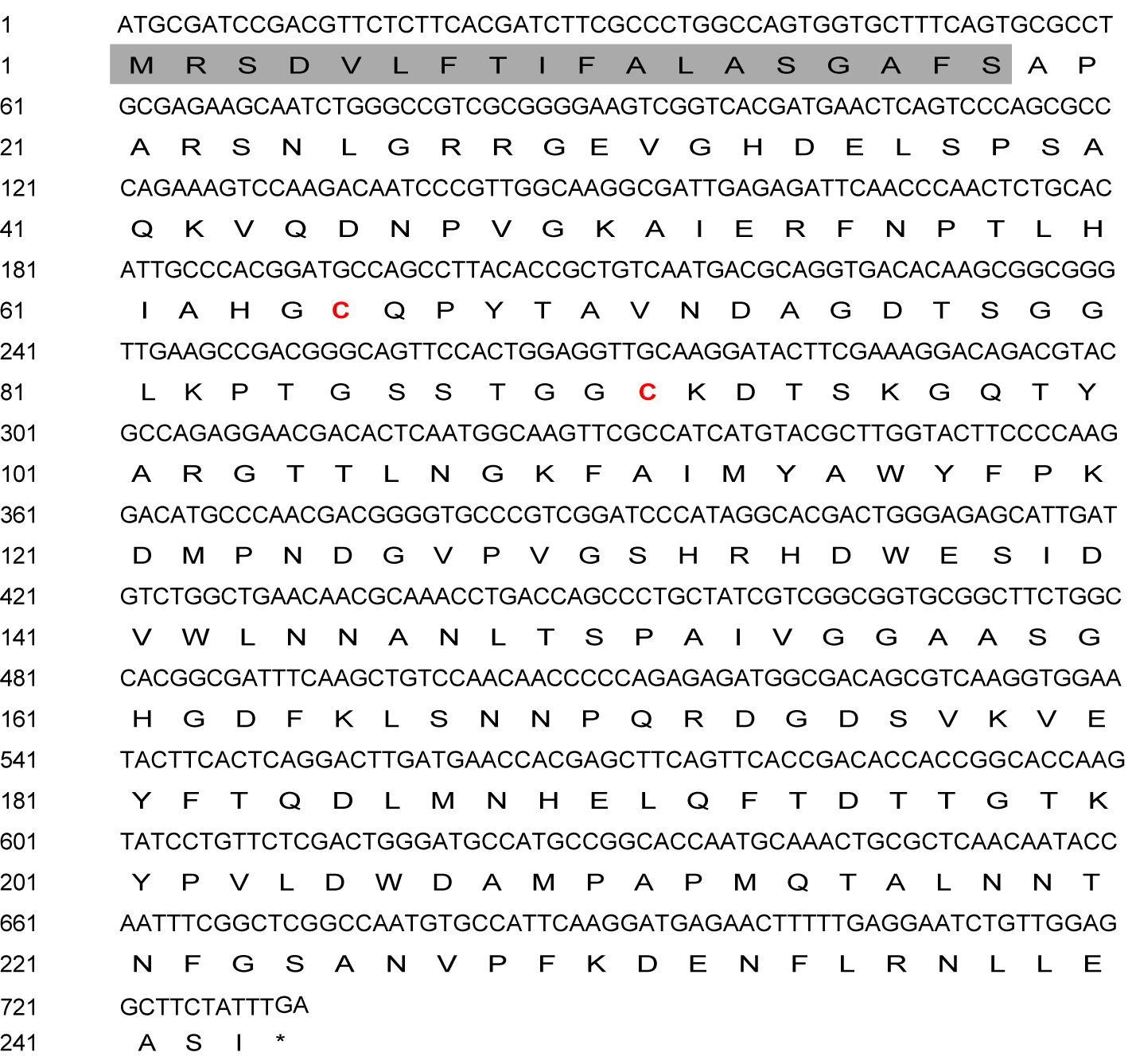

Supplement: Supplementary Figure S1 — Nucleotide sequence and deduced amino acid sequence of CgNLP1. Shading indicates the amino acid sequences of the signal peptide. [file Image_1.TIF]

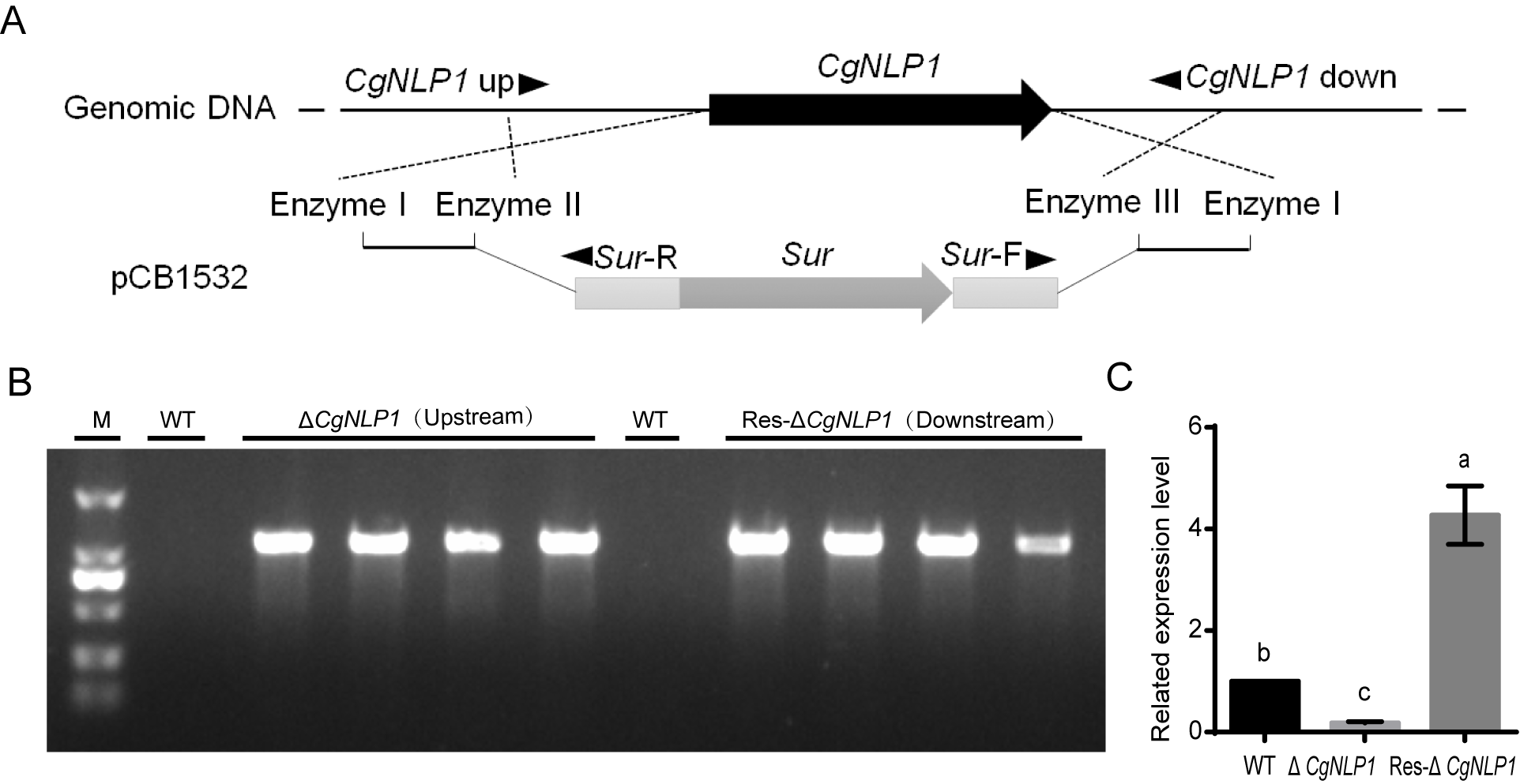

Supplement: Supplementary Figure S2 — Generation and molecular confirmation of CgNLP1 deletion mutant (ΔCgNLP1) and complementation mutant (Res-ΔCgNLP1). (A) The diagram of the CgNLP1 knockout vector. (B) Diagnostic PCR analysis for deletion of CgNLP1 and integration of CgNLP1 into the genome of C. gloeosporioides. (C) The expression level of CgNLP1 in wild type (WT), ΔCgNLP1, and Res-ΔCgNLP1 by quantitative RT-PCR. [file Image_2.TIF]

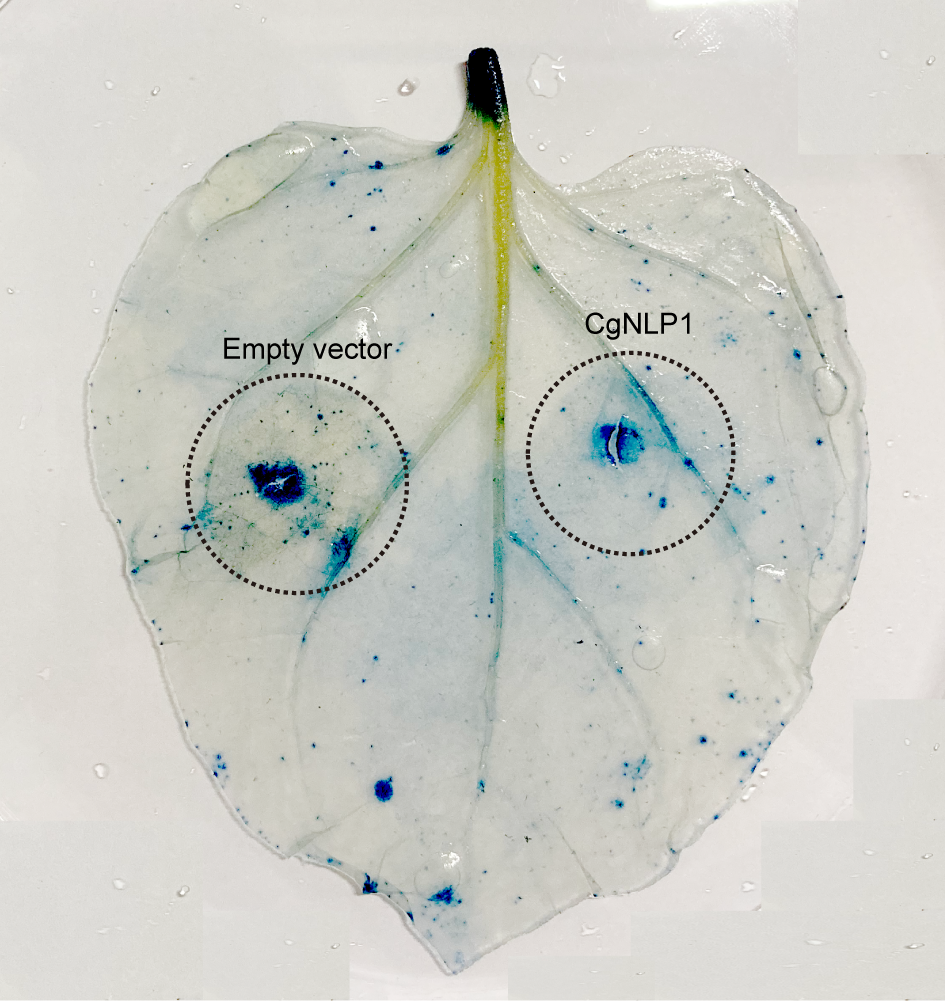

Supplement: Supplementary Figure S3 — Trypan blue transient of tobacco leaves expressing CgNLP1. pEGAD indicates the tissue expressing empty vector pEGAD-eGFP, and pEGAD-CgNLP1 indicates the tissue expressing recombinant vector pEGAD-CgNLP1-eGFP. [file Image_3.TIF]

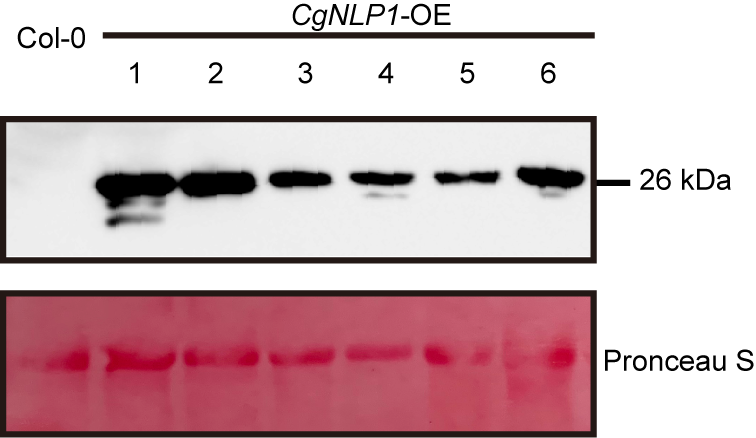

Supplement: Supplementary Figure S4 — Identification of CgNLP1 transgenic Arabidopsis lines by Western blot. Col-0 indicates Arabidopsis Columbia-0, and 1-6 indicates different CgNLP1-OE transgenic lines under the Col-0 background. [file Image_4.TIF]

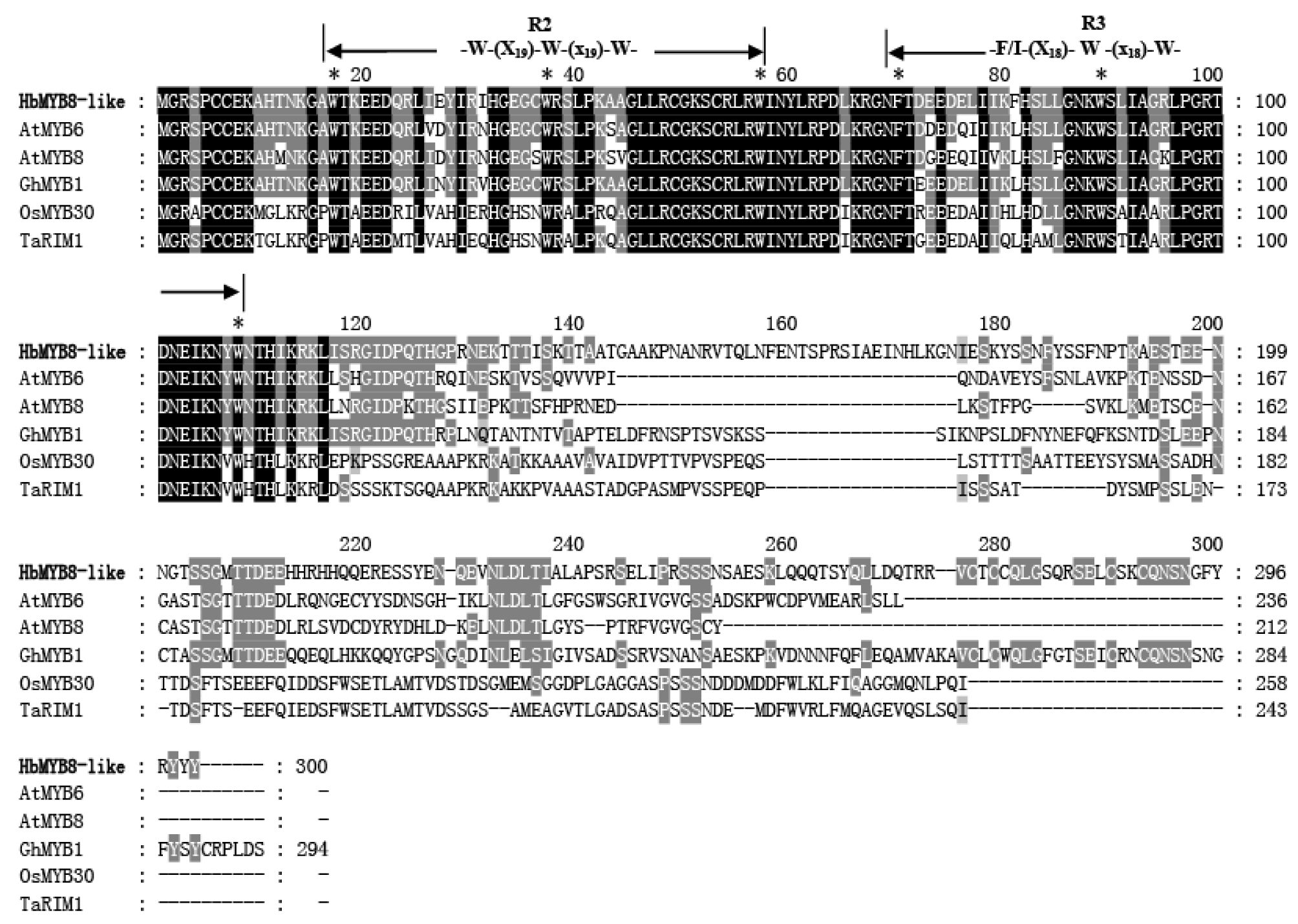

Supplement: Supplementary Figure S5 — Alignment of HbMYB8-like and homologs from different plants. Shading indicated regions of conservation in all (black), the same amino acid as HbMYB8-like (gray) sequences. The protein sequences used for alignment are as follows: Arabidopsis thaliana AtMYB6 (EFH48703.1), Arabidopsis thaliana AtMYB8 (Q9SDS8.1), Oryza sativa OsMYB30 (Q6K1S6.1), Gossypium hirsutum GhMYB1 (NP_001313761.1), and Triticum aestivum TaRIM1 (AMP18876.1). [file Image_5.TIF]

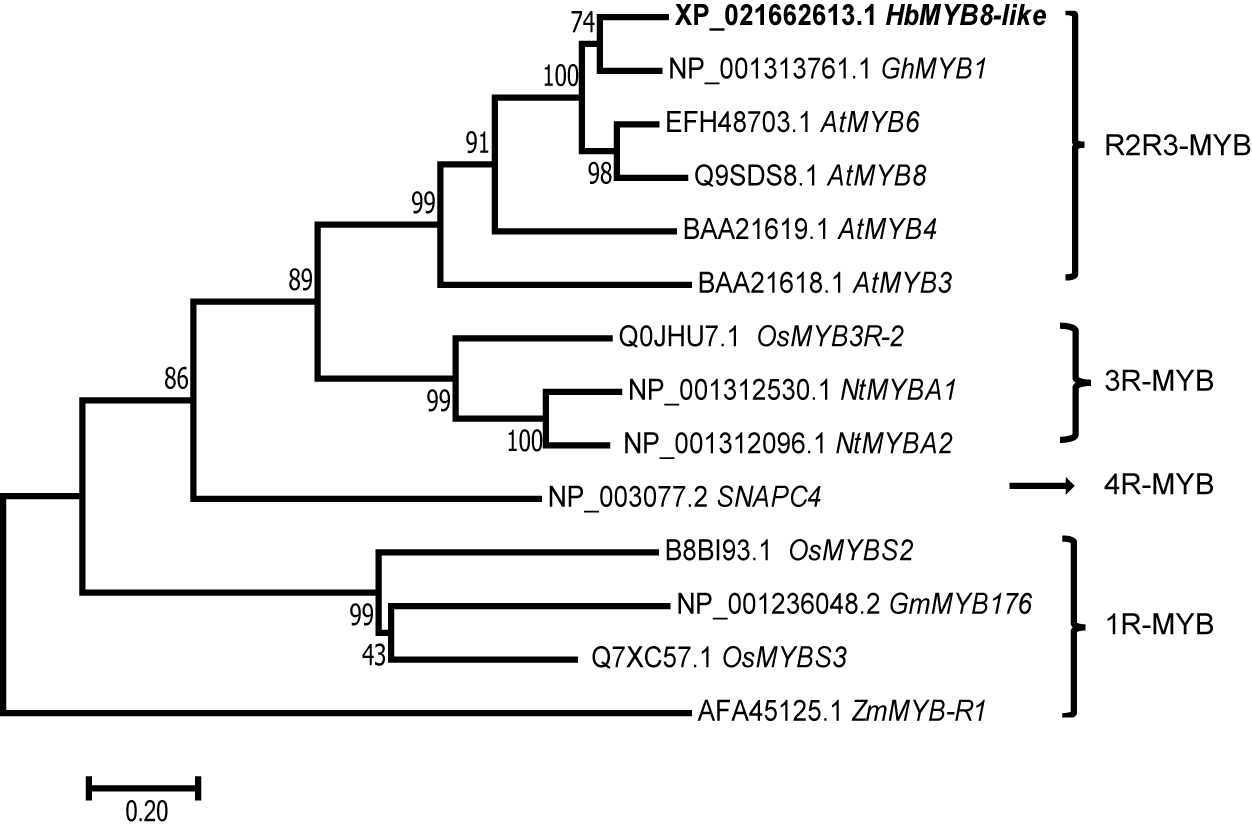

Supplement: Supplementary Figure S6 — Phylogenetic tree of HbMYB8-like with different types of MYB proteins in plants. [file Image_6.TIF]
